# 5-Aminolevulinic Acid Induced Fluorescence Is a Powerful Intraoperative Marker for Precise Histopathological Grading of Gliomas with Non-Significant Contrast-Enhancement

**DOI:** 10.1371/journal.pone.0076988

**Published:** 2013-10-18

**Authors:** Georg Widhalm, Barbara Kiesel, Adelheid Woehrer, Tatjana Traub-Weidinger, Matthias Preusser, Christine Marosi, Daniela Prayer, Johannes A. Hainfellner, Engelbert Knosp, Stefan Wolfsberger

**Affiliations:** 1 Department of Neurosurgery, Medical University Vienna, Vienna, Austria; 2 Institute of Neurology, Medical University Vienna, Vienna, Austria; 3 Department of Nuclear Medicine, Medical University Vienna, Vienna, Austria; 4 Department of Internal Medicine 1, Medical University Vienna, Vienna, Austria; 5 Department of Radiology, Medical University Vienna, Vienna, Austria; 6 Comprehensive Cancer Center – Central Nervous System Tumours Unit (CCC-CNS), Medical University Vienna, Vienna, Austria; Glasgow University, United Kingdom

## Abstract

**Background:**

Intraoperative identification of anaplastic foci in diffusely infiltrating gliomas (DIG) with non-significant contrast-enhancement on MRI is indispensible to avoid histopathological undergrading and subsequent treatment failure. Recently, we found that 5-aminolevulinic acid (5-ALA) induced protoporphyrin IX (PpIX) fluorescence can visualize areas with increased proliferative and metabolic activity in such gliomas intraoperatively. As treatment of DIG is predominantely based on histopathological World Health Organisation (WHO) parameters, we analyzed whether PpIX fluorescence can detect anaplastic foci according to these criteria.

**Methods:**

We prospectively included DIG patients with non-significant contrast-enhancement that received 5-ALA prior to resection. Intraoperatively, multiple samples from PpIX positive and negative intratumoral areas were collected using a modified neurosurgical microscope. In all samples, histopathological WHO criteria and proliferation rate were assessed and correlated to the PpIX fluorescence status.

**Results:**

A total of 215 tumor specimens were collected in 59 patients. Of 26 WHO grade III gliomas, 23 cases (85%) showed focal PpIX fluorescence, whereas 29 (91%) of 33 WHO grade II gliomas were PpIX negative. In intratumoral areas with focal PpIX fluorescence, mitotic rate, cell density, nuclear pleomorphism, and proliferation rate were significantly higher than in non-fluorescing areas. The positive predictive value of focal PpIX fluorescence for WHO grade III histology was 85%.

**Conclusions:**

Our study indicates that 5-ALA induced PpIX fluorescence is a powerful marker for intraoperative identification of anaplastic foci according to the histopathological WHO criteria in DIG with non-significant contrast-enhancement. Therefore, application of 5-ALA optimizes tissue sampling for precise histopathological diagnosis independent of brain-shift.

## Introduction

Decisions concerning postoperative adjuvant therapy of diffusely infiltrating gliomas (DIG) are based on the histopathological World Health Organisation (WHO) criteria: While in high-grade gliomas (WHO grade III and IV) immediate postsurgical radio- or radiochemotherapy is essential [1]–[3], in the majority of low-grade gliomas (WHO grade II) maximum safe tumor resection is currently regarded as first and only treatment until malignant progression develops [4]–[7]. This malignant progression generally originates within a circumscribed intratumoral area, the so called “anaplastic focus” [8], [9]. DIG are typically characterized by substantial intratumoral histological heterogeneity, which in many cases complicates accurate histopathological diagnosis [8]. If the anaplastic focus is missed during tissue sampling at open surgery or biopsy, histopathological undergrading of already high-grade gliomas results and – consequently – the patient will not receive the required postoperative adjuvant treatment [8], [10].

DIG with non-significant contrast-enhancement (CE) on magnetic resonance imaging (MRI) pose a special challenge to the neurosurgeon, as an unequivocal CE on MRI is not available as a reliable target for tissue sampling from the suspected anaplastic focus. To avoid sampling error in these tumors, navigation-guided tissue sampling from the anaplastic focus identified by metabolic imaging using positron emission tomography (PET) or MRI spectroscopy - chemical shift imaging (CSI) has been proposed [10]–[12]. However, brain-shift during the neurosurgical resection may cause navigational inaccuracy impeding precise tissue sampling [13]. Therefore, new and reliable intraoperative techniques for visualizing anaplastic foci that are independent of brain-shift need to be developed.

After oral administration, 5-aminolevulinic acid (5-ALA) leads to accumulation of intraoperatively visible fluorescing protoporphyrin IX (PpIX) predominantly in malignant glioma cells and is therefore widely used for fluorescence-guided resection of DIG with significant CE [14]–[16]. In a pilot study of 17 patients, we previously identified 5-ALA induced PpIX fluorescence as a promising marker for intraoperative detection of anaplastic foci in DIG with non-significant CE [17]. However, due to the small patient cohort, a systematic review of histopathological parameters according to the current WHO criteria in PpIX fluorescence positive versus negative tumor samples has not been performed.

The current study consists of a larger patient series designed to validate our previous results. As treatment decisions mostly depend on the histopathological diagnosis we furthermore accomplished the correlation of PpIX fluorescence status with histopathological parameters which are relevant for tumor grading.

The aim of the current study was therefore to clarify, whether focal PpIX fluorescence is able to visualize intratumoral areas in DIG with non-significant CE that correspond histopathologically to WHO grade III tumor tissue to optimize neurosurgical tissue sampling and enhance precision of histopathological glioma grading.

## Patients and Methods

Our present study comprises 59 patients with DIG with non-significant CE on MRI including the 17 patients of our pilot study.[17] All patients underwent tumor resection at the Department of Neurosurgery of the Medical University Vienna between March 2008 and July 2012. Patients had no prior history of treatment with chemo- or radiotherapy. This study was approved by the Medical University Vienna ethics committee (EC number: 419/2008) and all patients gave written informed consent to participate in this study. Patient characteristics are provided in *table 1*.

**Table 1:** Patient characteristics.

		*n*	*%*
**Number of patients**		**59**	**(100)**
**Gender**	female∶male	1∶1.2	
**Age**	median (range)	41 years (20–74)	
**Localization**	frontal	28	(47)
	temporal	14	(24)
	central	7	(12)
	insular	5	(8)
	parietal	3	(5)
	occipital	1	(2)
	thalamus	1	(2)
**MRI contrast enhancement**			
	none	24	(41)
	patchy/faint	19	(32)
	focal	16	(27)
**Extent of resection**	gross total	38	(64)
	partial	21	(36)
**WHO grade**	**Grade II**	**33/59**	**(56)**
Diagnosis	Diffuse astrocytoma	16	(27)
	Oligodendroglioma	9	(15)
	Mixed oligoastrocytoma	8	(14)
**WHO grade**	**Grade III**	**26/59**	**(44)**
Diagnosis	Anaplastic astrocytoma	9	(15)
	Anaplastic oligodendroglioma	10	(17)
	Anaplastic oligoastrocytoma	7	(12)

MRI = magnetic resonance imaging, WHO = World Health Organisation.

### Preoperative Imaging

Preoperative imaging (MRI, PET and/or CSI) was performed within two weeks prior to surgery:

#### Magnetic resonance imaging (MRI)

Our routine MRI protocol for intracranial tumors was performed on a 3 Tesla scanner (Tim Trio, Siemens, Erlangen, Germany) in all patients as described previously: Axial fluid-attenuated-inversion-recovery (FLAIR) sequences, diffusion-weighted images (DWI), axial, coronal T1- and T2-weighted sequences, contrast-enhanced axial, coronal and sagittal T1-weighted sequences and additional diffusion tensor imaging (DTI) and functional MRI (fMRI) where indicated [11], [17], [18].

**Figure 1:**
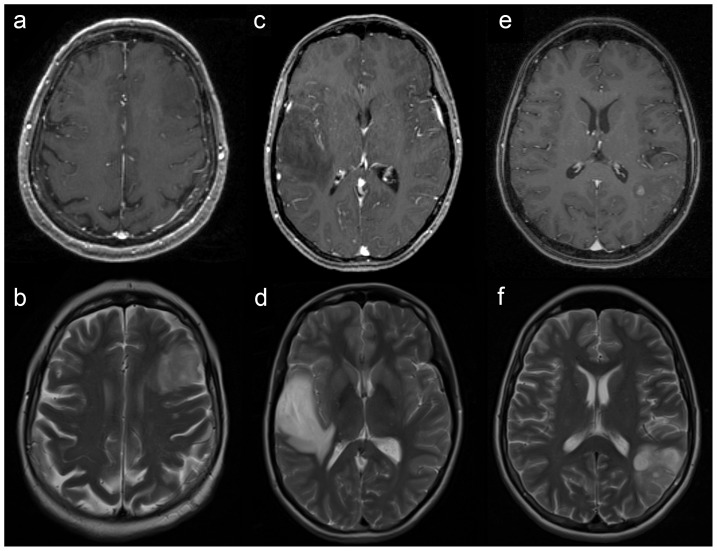
Pattern of contrast-enhancement (CE) on magnetic resonance imaging (MRI) of gliomas with non-significant CE. T1-weighted contrast-enhanced MR images demonstrate examples of gliomas with (a) no visible ( = *none*) CE, (c) unspecific ( = *patchy/faint*) CE and (e) a small regional ( = *focal*) CE in an otherwise non-enhancing tumor. (b,d,f) T2-weighted MR images show the corresponding hyperintense glioma lesions.

The presence of CE on MRI was assessed by an experienced neuroradiologist (D.P.):

1. DIG with non-significant CE: Only gliomas with non-significant CE on MRI that were initially defined by *none* or *patchy/faint* ( = unspecific) CE were included in the present study as described previously (see *Figure 1a+b*) [11], [17], [18]. As we observed since our pilot study an additional pattern of CE on MRI with a small circumscribed contrast-enhancing intratumoral area of malignant transformation that represents a potential target area for positive PpIX fluoresecence, we additionally included this category in the current imaging classification [17]. Consequently, we defined such contrast-uptake pattern as *focal CE* that is characterized by the presence of a small regional CE in an otherwise non-enhancing tumor (see *Figure 1c*).
2. DIG with significant CE: In contrast, patients with significant CE defined as *nodular* ( = homogeneous CE of a large portion or the entire tumor) or typical *ring-like* CE were excluded.

#### Positron emission tomography (PET)

PET was performed with an amino acid tracer using either ^11^C-methionine (MET; n = 45/59 patients) or ^18^F-fluoro-ethyl-L-tyrosine (FET; n = 10/59 patients) for detection of potential anaplastic foci in the majority of patients (n = 55/59 patients). Data acquisition and image reconstruction for MET-PET was performed as described previously [11], [17], [19]. FET was synthesized by ^18^F - fluoralkylation of tyrosine as described previously [20]. After a transmission scan for 5 minutes FET-PET started with tracer injection (mean activity 180–250 MBq). A dynamic acquisition was performed for 40 minutes (five 4–minute and two 10–minute frames). Scatter correction and reconstruction parameters were comparable to MET-PET. For visual image analysis and placement of regions of interest, FET-PET studies were summed between 20 and 40 minutes. In MET-PET as well as FET-PET, we used for tumor tissue sampling the area with the highest tumor to normal brain ratio (T/N ratio ≥1.2) defined as PET_max_ as biopsy target [11], [17].

#### Chemical Shift Imaging (CSI)

If PET did not detect an area of increased metabolism (T/N ratio <1.2) or was unavailable, the highest pathologic CSI ratio (choline/N-acetyl-aspartate ratio), defined as CSI_max_, was used as biopsy target for tumor tissue sampling as described previously [11].

### Administration of 5-ALA

Oral solutions of 5-ALA (20 mg/kg bodyweight Gliolan®; medac, Germany or Biosynth® AG, Switzerland) were administered three hours before anesthesia in all patients. Intraoperative visualization of PpIX fluorescence was performed using a modified neurosurgical microscope (NC4 and Pentero, Carl Zeiss Surgical GmbH, Oberkochen, Germany) with integrated violet-blue excitation light module [21]. All patients were protected from light sources for at least 24 hours after 5-ALA intake to prevent potential skin phototoxicity.

### Neurosurgical Glioma Resection

All glioma resections were performed with navigational guidance (Stealth Station Cranial Treon or S7; Medtronic, CO, USA) using T1-contrast-enhanced MRI co-registered with PET_max_ or CSI_max_ as described elsewhere [11], [17]. Depending on tumor location, additional functional imaging data from fMRI and DTI were used as appropriate. The following protocol was applied for tissue sampling and assessment of potential PpIX fluorescence during each glioma resection:

#### Sampling procedure and biopsy target selection

The biopsy target was approached at the beginning of the glioma resection through a small corticotomy to minimize the consequences of potential brain-shift as described previously [17]. Biopsy targets for tissue sampling were selected according to the following algorithm:

1. If PET detected an increased intratumoral metabolism (n = 44/59 cases; T/N ratio ≥1.2), PET_max_ was used as biopsy target.
2. If PET did not detect an increased intratumoral metabolism (n = 11/59 cases; T/N ratio <1.2), CSI_max_ (n = 5 cases) was used as biopsy target. If CSI was not available (n = 6 cases) multiple intratumoral samples were collected.
3. If PET was not available (n = 4/59 cases), CSI_max_ (n = 1 case) or focal CE on MRI (n = 3 cases) were used as biopsy target.

#### Tissue sampling outside the biopsy target

Subsequently, tissue sampling of multiple intratumoral areas outside the biopsy target was performed to confirm the ability of focal PpIX fluorescence for identification of anaplastic foci.

#### Assessment of PpIX fluorescence during glioma resection

During each tumor resection the PpIX fluorescence status was checked repeatedly with our modified neurosurgical microscope by switching between white-light and violet-blue excitation light in the different intratumoral areas starting at the biopsy target. The PpIX fluorescence status of each analysed intratumoral area was documented as PpIX positive or negative by the performing neurosurgeon and subsequently samples of these PpIX positive or negative intratumoral regions were collected for histopathological assessment. In case of positive PpIX fluorescence, additional topographical correlation of the PpIX positive intratumoral area with the biopsy target (PET_max_, CSI_max_ or focal CE) was performed by using our navigation system as described previously [17]. Finally, the remaing PpIX positive tumor tissue was removed if this was feasible from neurological point of view.

### Histopathology

In each glioma, the whole tumor specimen and all additional PpIX positive and negative glioma samples were formalin-fixed and paraffin-embedded and routinely processed for conventional histopathology.

#### Histopathological WHO diagnosis

For the histopathological diagnosis, the whole tumor specimen and all additional PpIX positive and negative samples of each glioma were histopathologically analyzed. The histopathological diagnosis of each glioma was established according to the WHO 2007 criteria with regard to tumor type and grade by the local neuropathology team (J.A.H., A.W.) on a multi-headed microscope [22]. The neuropathologists were blinded to the PpIX fluorescence status. A detailed distribution of diagnoses is provided in *table 1*.

#### Histopathological analysis of PpIX positive and negative samples

Furthermore, histopathological indicators of anaplasia according to the current WHO criteria were assessed in all collected PpIX positive and negative tumor specimens with the following semiquantitative grading system: (1) Tumor cell density (0 = low; 1 = moderate; 2 = high), nuclear pleomorphism (0 = low; 1 = moderate; 2 = high), mitotic activity (0 = no; 1 = few; 2 = many), presence of microvascular proliferation (0 = no; 1 = yes), and necrosis (0 = no; 1 = yes). Finally, proliferation rate was assessed in hot spots and expressed as percentage (MIB-1 labeling index (LI); anti-Ki-67, 1∶50; DAKO, Glostrup, Denmark) as previously described [11], [17].

#### Comparison of histopathological parameters

We performed a comparison of these histopathological parameters between PpIX positive and negative gliomas and PpIX positive and negative samples within the same tumor.

If more than one tissue sample was available per PpIX positive and/or negative glioma, the highest values of the histopathological WHO indicators of anaplasia and highest MIB-1 LI of each glioma were selected for further analysis [17].

### Statistical analysis

For statistical analyses SPSS® version 20.0 software (SPSS Inc., Chicago, Illinois, USA) was used. For correlation of PpIX fluorescence status with CE on MRI and histopathological WHO criteria the *X^2^*-test was used. MIB-1 and PET T/N values showed a right-skewed distribution. Therefore, for comparison of MIB-1 LI and T/N values between subgroups non-parametric tests (Mann-Whitney-U test for independent data and Wilcoxon rank-sum test for paired data) were used. A P-value of <0.05 was considered significant.

## Results

In the present study, 5-ALA was administered in 59 DIG patients with non-significant CE on MRI. In none of the cases any 5-ALA-related adverse effects were observed. During intraoperative tissue sampling, a total of 215 tumor specimens (median 3; range 1–13 specimens) were collected. Focal PpIX fluorescence was observed in 27 (46%) of the 59 gliomas, no visible PpIX fluorescence was detected in any intratumoral area of the remaining 32 cases (54%).

### PpIX fluorescence correlates with the amount of CE (see *table 2*)

**Table 2:** All gliomas of the current study (n = 59): Assessment of the amount of contrast-enhancement on magnetic resonance imaging, WHO tumor grade, histopathological WHO criteria and proliferation rate in PpIX focally positive versus negative gliomas.

		*PpIX fluorescence*				
*all tumors n = 59*		*negative*		*Focally positive*		*p*
		*tumors*		*tumors*		
Patients	n	32	(100%)	27	(100%)	
MRI CE	none	21	(66%)	3	(11%)	<0.0001
	patchy/faint	9	(28%)	10	(37%)	
	focal	2	(6%)	14	(52%)	
WHO grade	grade II	29	(91%)	4	(15%)	<0.0001
	grade III	3	(9%)	23	(85%)	
Histopathological criteria						
Mitosis	absent	29	(91%)	2	(7%)	<0.0001
	few	3	(9%)	14	(52%)	
	many	0	(0%)	11	(41%)	
Cell density	low	14	(44%)	1	(4%)	0.001
	moderate	15	(47%)	17	(63%)	
	high	3	(9%)	9	(33%)	
Nuclear	low	21	(66%)	2	(7%)	<0.0001
pleomorphism	moderate	9	(28%)	16	(60%)	
	high	2	(6%)	9	(33%)	
Microvascular	no	32	(100%)	24	(89%)	n.s.
proliferation	yes	0	(0%)	3	(11%)	
Necrosis	no	32	(100%)	26	(96%)	n.s.
	yes	0	(0%)	1	(4%)	
MIB-1 (%)	mean ± SD	6.9±5.4		15.9±8.4		<0.0001

MR CE = magnetic resonance imaging contrast-enhancement.

We found a positive correlation of visible PpIX fluorescence with the intensity of CE on preoperative MRI: In the 24 patients with no CE on MRI, positive PpIX fluorescence was detected in only 13% of cases. In the 19 patients with patchy/faint CE on MRI, visible PpIX fluorescence was observed intraoperatively in 53% of cases. Finally, the majority of gliomas with focal CE were PpIX positive, namely 88% of 16 patients. This difference was statistically significant (p<0.0001).

### PpIX fluorescence correlates with PET, CSI, and focal CE (*see* *figure 2*)

**Figure 2:**
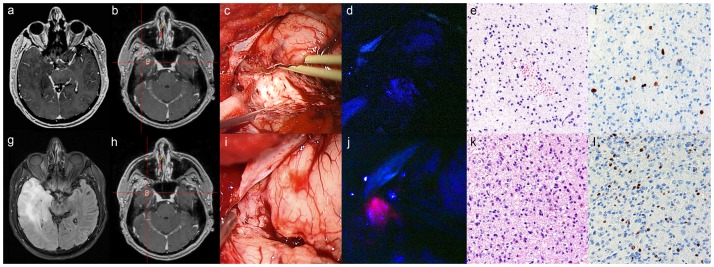
Example of 5-ALA application in a left temporal glioma with non-significant contrast-encencement (CE) on magnetic resonance imaging (MRI). (a) Preoperative contrast-enhanced T1-weighted MR images show patchy/faint CE and (g) hyperintensity on FLAIR sequences. (b) The intratumoral area outside the region of maximum positron emission tomography (PET) tracer uptake verified by the intraoperative navigation system (c) appeared as whitish glioma tissue under the surgical microscope, (d) with no detectable PpIX fluorescence. (e) The corresponding histopathology reveals low-grade glioma tissue according to the WHO criteria in the H&E stain (f) with a low proliferation rate (MIB-1: <10%). (h) In contrast, the intratumoral area inside the region of maximum PET tracer uptake (i) showed similar glioma tissue appearance in the microscopic view, (j) but revealed strong PpIX fluorescence under violet-blue excitation light. (k) The corresponding histopathology reveals high-grade glioma tissue in accordance with an anaplastic focus according to the WHO criteria in the H&E stain (l) with a high proliferation rate (MIB-1: 32%). The final histopathological diagnosis revealed a focally anaplastic astrocytoma (WHO grade III) and the patient was treated with radiochemotherapy. The width of each histopathological image (e, f, k, l) was 300 micrometers (µm).

In all 27 gliomas with positive PpIX fluorescence, the fluorescence effect was only detected in one specific region of the glioma, all other intratumoral areas were PpIX negative. In all of these patients revealing a distinct PET_max_ (n = 23/27 cases), focal PpIX fluorescence correlated topographically with the biopsy target area of PET_max_. In absence of a distinct PET_max_ (n = 1) or PET imaging was not available (n = 3), focal PpIX fluorescence was detected in the biopsy target area of focal CE (n = 3) and CSI_max_ (n = 1). Additionally, we investigated the PET tracer uptake in focally PpIX positive versus negative gliomas: The T/N ratio was significantly higher in the focally PpIX positive as compared to the PpIX negative group (T/N ratio 2.2±0.7 versus 1.6±0.6; p = 0.002).

### PpIX fluorescence correlates with WHO tumor grading (see *table 2* *and* *figure 2*)

The PpIX fluorescence status correlated with tumor grading according to the WHO criteria: Of the 26 gliomas corresponding to WHO grade III, 23 gliomas (85%) revealed focal positivity of PpIX fluorescence intraoperatively. In contrast, of the 33 WHO grade II gliomas, 29 cases (91%) were PpIX negative. This difference was statistically significant (p<0.0001). The sensitivity of focal PpIX fluorescence for WHO grade III histology was 88%, the specificity was 89%. For details see *table 3*.

**Table 3:** Predictive relationship of visible PpIX fluorescence and anaplastic histology.

Factor	Anaplastic histology	
	%	(95% CI)
sensitivity	89%	(69–97)
PPV	85%	(65–95)
specificity	88%	(71–96)
NPV	91%	(74–98)

PPV = positive predictive value; NPV = negative predictive value; CI = confidence interval.

### PpIX fluorescence correlates with histopathological WHO criteria of anaplasia and increased proliferation rate (*see* *figure 2*)

#### Focally PpIX positive tumors versus PpIX negative tumors (see table 2)

Histopathological WHO criteria for anaplasia were compared between the group of focally PpIX positive gliomas and PpIX negative gliomas (n = 59 patients): In focally PpIX positive gliomas, mitotic rate (p<0.0001), cell density (p = 0.001) and nuclear pleomorphism (p<0.0001) were significantly higher as compared to PpIX negative gliomas. Furthermore, the proliferation index assessed by MIB-1 LI was significantly higher in focally PpIX positive gliomas in comparison to tumors with no visible fluorescence (mean MIB-1 LI: 15.9 versus 6.9%; p<0.0001).

#### Focally PpIX positive areas versus PpIX negative areas (see table 4)

In the focally PpIX positive gliomas, histopathological WHO parameters were compared between paired samples of the focally PpIX positive areas and negative areas within the same tumor (n = 27 patients): In focally PpIX positive areas, mitotic rate (p<0.0001), cell density (p = 0.014) and nuclear pleomorphism (p = 0.001) were significantly higher as compared to PpIX negative areas. Microvascular proliferations and necrosis were only exceptionally present in single anaplastic oligodendroglial tumors (see *table 4*), all of which displayed PpIX fluorescence. Additionally, MIB-1 LI was significantly higher in focally PpIX positive versus negative areas within the same tumor (mean MIB-1 LI: 15.9 versus 8.8%; p<0.0001).

**Table 4:** All PpIX fluorescence positive tumors (n = 27): Assessment of histopathological WHO criteria and proliferation rate in PpIX focally positive versus negative intratumoral areas within the same glioma.

*all PpIX fluorescence*		*PpIX fluorescence*				
*positive tumors*		*negative*		*focally*		*p*
*n = 27*		*area*		*positive*		
Mitosis	no	16	(59%)	2	(7%)	<0.0001
	few	11	(41%)	14	(52%)	
	many	0	(0%)	11	(41%)	
Cell density	low	8	(30%)	1	(4%)	0.014
	moderate	16	(59%)	17	(63%)	
	high	3	(11%)	9	(33%)	
Nuclear	low	15	(56%)	2	(7%)	0.001
pleomorphism	moderate	9	(33%)	16	(60%)	
	high	3	(11%)	9	(33%)	
Microvascular	no	27	(100%)	24	(89%)	n.s.
proliferation	yes	0	(0%)	3^a^	(11%)	
Necrosis	no	27	(100%)	26	(96%)	n.s.
	yes	0	(0%)	1^b^	(4%)	
MIB-1 (%)	mean ± SD	8.8±5.2		15.9±8.4		<0.0001

a Two anaplastic oligodendrogliomas and one anaplastic oligoastrocytoma,

b One anaplastic oligodendroglioma.

## Discussion

In the present study, we investigated the clinical relevance of 5-ALA induced PpIX fluorescence for intraoperative identification and tissue sampling of anaplastic foci to increase precision of histopathological tumor grading of DIG with non-significant CE. In our series of 59 diffuse glioma patients, we found a significant correlation of focal PpIX fluorescence with histopathological WHO indicators of anaplasia indicating the capability of 5-ALA to accurately detect anaplastic foci within DIG.

### Application of 5-ALA in neurosurgery

In malignant gliomas with significant CE, Stummer et al. were able to demonstrate in their randomized controlled multicenter phase 3 trial that 5-ALA fluorescence-guidance resulted in a significantly higher rate of complete surgical removal of the contrast-enhancing tumor and a significantly prolonged 6-month progression-free survival as compared to the control group [14]. According to a lately published literature review and meta-analysis of relevant prospective studies there exists level 2 evidence that 5-ALA fluorescence-guided resections of high-grade gliomas are more effective than conventional white-light procedures [16]. Recently, visible PpIX fluorescence was also reported in other tumor entities such as meningiomas and metastases after 5-ALA application [23]–[27]. Lately, our group identified strong 5-ALA induced protoporphyrin IX fluorescence as an immediate available intraoperative marker for representative tumor tissue especially of malignant gliomas and intracranial lymphomas – a finding also observed by others [18], [28], [29]. In DIG with non-significant CE, however, the value of 5-ALA application remained yet to be elucidated.

### Pilot study: 5-ALA for detection of anaplastic foci unrelated to brain-shift

Sampling of the anaplastic focus during resection of DIG with non-significant CE is prone to error: Surgical targeting is usually performed with navigational guidance that relies on preoperative metabolic image information such as PET and/or CSI [10]–[12]. Increasing brain-shift in the course of the tumor resection, however, leads to progressive navigational inaccuracy [13]. Updating the navigation system by intraoperative MRI is one solution to overcome brain-shift, but is costly, time consuming and is therefore not widely available [30], [31].

To evaluate 5-ALA induced PpIX fluorescence as an alternative marker for anaplastic foci within DIG, we administered 5-ALA to patients with non-significant CE on MRI in a pilot study [17]. Remarkably, we were able to detect visible PpIX fluorescence within a distinct intratumoral area in a fraction of these diffuse gliomas. In this pilot study of 17 diffuse gliomas, we observed a significant correlation of focal PpIX fluorescence with the diagnosis of a high-grade glioma: We found focal PpIX fluorescence in 8 of 9 WHO grade III gliomas, whereas all 8 WHO grade II gliomas were PpIX negative. Additionally, histopathological assessment revealed a significantly higher cell proliferation rate in intratumoral areas with focal PpIX fluorescence as compared with PpIX negative areas within the same tumor.

### Application of 5-ALA in a large series of DIG with non-significant CE

However, treatment of glioma patients is not only based on the tumor proliferation rate, but includes other histopathological parameters such as cell density and nuclear pleomorphism as described by the current WHO criteria [22]. In DIG with non-significant CE, however, the histopathological parameters according to the WHO classification in focal PpIX positive versus negative tumor samples has not been systematically assessed. Based on our preliminary findings, we designed the present study to clarify if regions of focal PpIX fluorescence in DIG with non-significant CE correspond histopathologically to anaplastic tumor areas according to the current WHO criteria.

### Correlation of focal PpIX fluorescence with diagnosis of a high-grade glioma

According to our results in the present large series of 59 gliomas, most WHO grade III gliomas (23 of 26 patients) showed visible PpIX fluorescence in a distinct intratumoral region, whereas the majority of WHO grade II gliomas (29 of 33 patients) did not. Thus, our findings indicate that 5-ALA induced PpIX fluorescence is able to detect anaplastic tumor tissue with high sensitivity and specificity (89% and 88%, respectively). Accordingly, we found in a recent series of stereotactic brain tumor biopsies that the vast majority of samples of WHO grade III and IV gliomas revealed strong 5-ALA induced PpIX fluorescence at the target region, whereas all specimens of WHO grade II gliomas were PpIX negative [18]. Similarly, Ewelt et al. detected visible PpIX fluorescence in 12 of 17 (70.6%) WHO grade III and IV gliomas in the region of the biopsy “hotspot”, whereas only one of 13 WHO grade II gliomas showed positive PpIX fluorescence [32]. Therefore, these independent observations including our current study provide strong evidence that 5-ALA induced PpIX fluorescence is predominantely present in high-grade gliomas.

### Correlation of focal PpIX fluorescence with histopathological signs of anaplasia

In this first systematic analysis of PpIX positive versus negative specimens in DIG with non-significant CE, we found significantly higher cell density, nuclear pleomorphism and mitotic rate not only in the group of focally PpIX positive as compared with negative gliomas, but also in focally PpIX positive as compared to PpIX negative tissue samples within the same tumor. Accordingly, Arita et al. observed a significantly higher cell density in fluorescence-positive than in fluorescence-negative samples at the periphery of mainly high-grade gliomas [33]. Furthermore, Roberts et al. found a strong correlation between positive PpIX fluorescence and a histopathological score that was based on the WHO criteria in 11 patients with newly diagnosed glioblastoma [34]. Additionally, we observed a significantly higher proliferation rate in focally PpIX positive as compared to PpIX negative gliomas and focally PpIX positive versus negative samples within a given glioma, which confirms the findings of our pilot study in a larger series. In agreement with our findings, recent *ex-vivo* and *in-vivo* studies reported a significantly higher proliferation rate in PpIX positive than negative tissue samples in gliomas [33], [35]–[37].

In sum, our results indicate that PpIX fluorescence is capable to identify anaplastic foci according to the WHO histopathological criteria. Therefore, we propose the application of 5-ALA for resection of radiologically suspected LGG additionally to the established imaging techniques (MRI, PET and CSI). While the latter techniques can be used for crude localization of the hotspot with a navigation system, positive PpIX fluorescence is subsequently able to exactly identify this area unaffected by brain-shift.

By using this approach, reliable tissue sampling from the anaplastic focus is possible to avoid histopathological undergrading. As we observed focal PpIX fluorescence especially in gliomas with focal and patchy/faint CE, such gliomas seem particularly good candidates for application of 5-ALA.

### Outlook

The vast majority of low-grade gliomas in our study did not reveal visible PpIX fluorescence in any intratumoral area. This observation is in accordance with reports of other groups [32], [37]–[41]. Recently, other groups were able to detect significant PpIX accumulation even in non-fluorescing low-grade glioma tissue by using either quantitative measurements of 5-ALA induced PpIX concentrations or confocal microscopy [36], [38], [42]. Additionally, Valdes et al. were able to demonstrate a significant linear correlation between PpIX concentrations in glioma tissue and quantitative histopathologic parameters indicating the potential of this new technique for detection of anaplastic foci especially in those cases with no visible PpIX fluorescence [36]. However, these techniques are currently not widely available in contrast to our proposed semiquantitative method.

Futhermore, the development of innovative tumor-specific fluorescent dyes or particular nanoparticles is crucial for detection of PpIX negative neoplasms [43]–[47]. These new techniques may be in future capable to visualize low-grade glioma tissue as well.

## Conclusion

Our study indicates that 5-ALA induced PpIX fluorescence is a powerful and clinically reliable marker for intraoperative identification of anaplastic foci according to the histopathological WHO criteria in DIG with non-significant CE. Consequently, application of 5-ALA optimizes intraoperative tissue sampling for precise histopathological grading to avoid undergrading and subsequent treatment failure independent of brain-shift.
